# Chunking in simultaneous interpreting: the impact of task complexity and translation directionality on lexical bundles

**DOI:** 10.3389/fpsyg.2023.1252238

**Published:** 2023-08-31

**Authors:** Dan Feng Huang, Fang Li, Hang Guo

**Affiliations:** ^1^School of Foreign Languages, Guangzhou College of Commerce, Guangzhou, Guangdong, China; ^2^School of Foreign Languages, Sun Yat-Sen University, Guangzhou, Guangdong, China; ^3^International College, Guangzhou College of Commerce, Guangzhou, Guangzhou, China

**Keywords:** simultaneous interpreting, lexical bundles, cognitive load, task complexity, directionality

## Abstract

This study explored the use of phraseological frames (p-frames), a type of lexical bundle, by simultaneous interpreters as a strategy for managing cognitive loads. Specifically, using a comparable corpus of United Nations Security Council conferences, the study employed kfNgram to automatically identify the p-frames, and investigated their variations, regarding frequency, fixedness, structures, and functions among L1, L1–L2, and L2–L1 texts, which differ in cognitive loads due to task complexity and directionality of interpreting. The findings indicated that interpreters used more p-frames as cognitive loads increased; No significant difference was identified in fixedness as all texts tended to employ heavily formulaic and relatively fixed p-frames. Directionality correlated with grammatical preferences, with retour interpreting relying more on content-word-based p-frames. Additionally, task complexity correlated with functional preferences, with self-expression characterized by more stance expressions in the simple task. This study innovatively addressed the interaction of two factors that generate cognitive loads in interpreting and filled a research gap by providing empirical evidence on how directionality affects the use of formulaic language.

## 1. Introduction

Chunking is an essential cognitive strategy for interpreters to cope with their cognitive loads at work. In interpreting, cognitive loads refer to the extent to which a task utilizes the human cognitive system, which has inherent limitations in its capacity (Seeber, 2013). Interpreters work under a heavy cognitive load, which can deteriorate their performance (Gile, 2009). The cognitive load approach proposed by Seeber (2011) attributed the high cognitive load in simultaneous interpreting (SI) to the real-time combination of comprehension and production. To ease the cognitive loads, interpreters implement the “law of least effort” (Gile, 2009, p. 213). Chunking is a cognitive strategy that interpreters use to implement the least effort law (Wu and Liao, 2018). By clustering information-carrying units, chunking facilitates comprehension and production in interpreting.

Formulaic sequences (FS) are linguistic units that realize chunking (Ellis, 2003), and interpreters can use them to manage cognitive loads. FS are a subset of phraseological units, which include “idioms, collocations, and sentence frames,” as defined by Wray (2000, p. 263). Appropriate use of these phraseological units can “buy time” and “create a shorter processing route” in speech production (Wray, 2000, p. 478), thereby facilitating interpreting. Researchers have recognized the advantages of using FS to aid interpreting since the last century (Ilg, 1985; Jones, 1998).

Corpus-based interpreting studies provide empirical evidence for the analysis of FS in interpreting. These studies have examined FS in connection with various factors that may overload interpreters cognitively. For instance, some studies have investigated the interpreting strategies that are required to overcome cross-linguistic differences (Li and Halverson, 2020; Wu et al., 2021; Li and Halverson, 2022), while some have explored the dual task requirement of comprehension and production in interpreting (Plevoets and Defrancq, 2018). Additionally, research has looked at how genres and registers impact the use of FS (Biel et al., 2019; Wu, 2021) and how interpreting in deficient environments affects FS usage (Song, 2020). Furthermore, insufficient interpreting experience has been studied concerning the use of FS (Wang and Huang, 2011, 2013; Tang and Jiang, 2022).

While these studies have confirmed the significance of FS in interpreting, they have also examined cognitive loads from multiple perspectives of interpreting tasks, environments, and interpreters. Importantly, they have provided insight into how interpreters flexibly use the chunking strategy to manage different types of cognitive loads. However, these studies have only considered a single factor of cognitive loads and have yet to adopt a multivariate perspective to measure cognitive loads. Interpreting is a multitasking and cognitively complex activity (Seeber, 2011; Lv and Liang, 2019; Stachowiak-Szymczak, 2019), and the interaction of different factors may shed light on this cognitive complexity. Connecting FS with the interactive effects of cognitive loads can yield a deeper understanding of their role in releasing cognitive loads.

Investigating FS use in a comparable corpus of conference interpreting (CI) provides a unique opportunity to explore the interaction between task complexity and directionality. Two primary modes of CI exist. They are SI and consecutive interpreting (Gile, 2008; Liang et al., 2019). In CI, L1 texts^1^, L1–L2, and L2–L1 interpreted texts exist simultaneously, presenting an opportunity to compare non-interpreted and interpreted texts, which incorporates task complexity, and to distinguish cognitive loads between L1–L2 and L2–L1 interpreting based on interpreting directionality (Chou et al., 2021).

The present study provided a multivariate perspective on cognitive loads in interpreting. It examined the variations of FS among L1, L1–L2, and L2–L1texts in a comparable corpus of CI and correlated these variations with cognitive loads caused by task complexity and directionality. The findings revealed how these two factors combined to affect the interpreters’ use of FS and provided insights into how interpreters employed the cognitive mechanism of chunking to cope with their cognitive loads, which has been considered a “more technical question” of CI (Gile, 2008, p. 53). Overall, this study contributed to a deeper understanding of the role of FS in managing cognitive loads.

## 2. Literature review

The current section first highlights the cognitive advantage of chunking in interpreting. It then moves on to discuss the use of formulaic sequences (FS) to implement chunking strategies. A synthesis of previous studies on FS illustrates the research gap to be addressed: none have yet looked at the interaction of potential factors in cognitive loads and tested the effect on FS. A multivariate perspective is proposed to fill this research gap.

### 2.1. Chunking and its advantages in interpreting

Chase and Simon (1973) were the first to note the cognitive advantage of chunking. They developed their chunking theory based on the study of expert chess players. According to the theory, expert players could recognize larger chunks of the knowledge of chess playing, enabling them to respond more quickly and appropriately.

Miller (1994) highlighted the limited range of WM capacity (which he postulated as “7 ± 2”) to illustrate the advantage conferred by chunking. Individuals, he pointed out, can only process a limited amount of information at any given time, and information-carrying units beyond the range may cause a cognitive load on WM. Grouping information in chunks can reduce the units for the same amount of information held in WM and thus relieve cognitive loads.

Cowan (2001) advanced a similar opinion: chunks are helpful as a measurement to cope with the cognitive loads on WM. WM shifts between different hierarchical levels of chunks to use limited units to hold the needed information and reduce its informative or cognitive loads. Additionally, chunks can be retrieved and merged into a larger chunk, further freeing up the limited capacities of WM.

The cognitive advantage of chunking operates across various kinds of information processing, such as human learning (Gobet et al., 2001), including the learning specifically referring to language learning (Ellis, 2003), developing motor skills (Diedrichsen and Kornysheva, 2015; Thompson et al., 2017), managing social information (Thornton and Conway, 2013), perceiving and producing speeches (Bonhage et al., 2014; Segawa et al., 2019) and interpreting (Gile, 2009; Seeber, 2011; Wu and Liao, 2018).

Chunking is a crucial strategy in interpreting. According to the effort models proposed by Gile (2009, p. 183), three types of efforts, namely the listening and analysis effort, the production effort, and the memory effort, require interpreters to “work close to their maximum capacity,” which is vividly described as the “Tightrope Hypothesis” of SI (Gile, 2009, p. 182). Cognitive overload occurs when the demands on a person’s cognitive resources exceed their capacity to handle them effectively. To understand the interpreters’ tactics and behaviors in coping with such overload, Gile constructed five prevailing laws. One of these he termed “the law of least effort”: using less time and capacities in processing information.

Wu and Liao (2018, p. 196) proposed that chunking “conforms to the norm of minimizing effort,” which is Gile’s other formulation of the law of least effort. They believed the benefit of chunking exists in both decoding and encoding meanings. In decoding meanings, chunking facilitates the synchrony between the source and target texts, called “time lag.” In encoding meanings, interpreters implement the chunking strategy to produce “short, clear, complete and easy to understand” (Wu and Liao, 2018, p. 197) output, mitigating the cognitive loads caused by interpreting in a weaker B language.

Seeber (2011, p. 189) was another scholar to suggest chunking as a strategy for management of SI. Seeber criticized the effort models for providing “a tenuous link” between comprehension and production in SI. Instead, he developed a cognitive load approach to better illustrate what causes cognitive loads during the dual tasks of synchronous comprehension and production. According to this approach, there are several reasons for heavy cognitive loads during SI. These include: overlap between the dual tasks, combinations of linguistic features, directionality, the symmetry between input and output, real-time processing, or combinations of these factors. Cognitive load management can be done at both macro and micro levels. Chunking operates at the micro level: It allows interpreters to decode meanings “without having to wait for the entire sentence to unfold” (Seeber, 2011, p. 194) and thus speed up meaning decoding.

Besides the above theoretical discussions, Ma and Li (2021) provided empirical evidence for the advantage of chunking in meaning decoding. In their study, two languages that cause word-order asymmetry in sight translation were considered to cause cognitive loads, which could be measured by eye-tracking. In coping with the asymmetry, interpreters could use the chunking strategy: Segmenting long sentences in the source text and presenting the segments in the target text. Evidence from eye-tracking indicated interpreters experienced less cognitive loads when they implemented chunking.

Given the cognitive advantage of chunking in reducing effort in interpreting, a sufficient application of FS, the lexical chunks, would therefore seem particularly desirable for interpreters. FS prevail in speech production and offer processing efficiency (Pawley and Syder, 1983). They help speakers fluently convey their meanings under time constraints (Kuiper and Haggo, 1984). Working under considerable time constraints is what makes interpreting cognitively challenging: interpreters have to “make decisions instantly, with strategies aimed at doing the best they can with what they have understood on the spot and under cognitive pressure” (Gile, 2008, p. 52). An in-depth understanding of how interpreters use FS helps reveal how they use chunking strategies to process cognitive loads.

### 2.2. Advantage of formulaic sequences in interpreting

Researchers have perceived multiword sequences in various ways and have also named them differently. Nattinger and DeCarrico (1992) termed co-occurring words lexical phrases, further dividing such phrases into four types: polywords, institutionalized expressions, phrasal constraints and sentence builders. They highlighted two features of lexical phrases: their use as lexico-grammatical units and their association with particular discourse functions. Moon (1997) used the term lexical chunks, referring to vocabulary consisting of a sequence of two or more words that semantically or syntactically form meaningful or inseparable units. O'Keeffe et al. (2007, p. 64) also used the term lexical chunks but emphasized the chunks’ pragmatic functions: lexical chunks “display pragmatic integrity and meaningfulness regardless of their syntax or lack of semantic wholeness.” Wray (2000, p. 463) adopted formulaic sequences (FS) as a subordinate term to cover various types of multiword units, including “idioms, collocations and sentence frames.” Later, Wray (2002) listed more than 50 terms to describe the phenomenon of FS, such as chunks, collocations, multiword units, and conventionalized forms. Recently, Lin made a concise summary of typical FS:

“FE is an umbrella term subsuming all types of lexicalized word combinations including idioms (e.g., *raining cats and dogs*, *spill the beans*), speech formulae (e.g., *easy does it*, let us *get on top on it*), proverbs (e.g., *an apple a day keeps the doctor away*), sayings (e.g., *better safe than sorry*, *time and tide wait for no man*), similes (e.g., *as white as snow*, *sleep like a log*), binomials (e.g., s*afe and sound*, *hustle and bustle*, *kith and kin*), collocations (e.g., a*sk-question*, *staggering-figures*, *prime minister*), and so on.” (Lin, 2022, p. 2).

Biber et al. (2004) used a corpus frequency-driven approach to retrieve multi-word sequences, and called them lexical bundles (LBs). Although LBs are not idiomatic, they are perfricated units and serve as a discourse framework to reflect chunking in the mental lexicon (Biber and Barbieri, 2007). LBs, when used appropriately, can facilitate communication for various purposes (Schmitt and Carter, 2004; Wood, 2010). With the advancement of corpus-based tools (e.g., kf-Ngram, AntGram, Concgrams), LBs can have various lengths (n-gram). Furthermore, p-frames were proposed to solve the overlap problem in the analysis of continuous LBs (Li et al., 2020). P-frames are discontinuous co-occurring LBs with one or more slots for variances (Lu, 2021). Overlap occurs quite often in continuous LBs. For example, there is an overlap between *it is found that* and *it was found that*. The overlap can “inflate the frequency of bundle instances” (Li et al., 2020, p. 88). A p-frame it * found that is extracted to resolve the overlap.

The current study assumes any type of multiword units stored in interpreters’ mind serve as holistic units that interpreters can use to implement a chunking strategy. It aims to draw a bigger picture of formulas in interpreting. Therefore, the term FS, which is “all-encompassing, covering a wide range of phraseology” (Schmitt and Carter, 2004, p. 4), is used to refer to phraseological use in interpreting.

Interpreters could turn to FS to save effort. Aston (2018) argued that professional interpreters relied on formulaic phraseologies to maintain fluency and ease their mental loads at work. Empirical evidence was gained through interpreting training. Student interpreters were able to anticipate more accurately what they were going to listen to and generate output more fluently if they prepared relevant FS in advance (Yang, 2018; Song, 2020). Early in the last century, effort was made to accumulate useful FS for interpreters (Ilg, 1985). Interest in the process of using FS in interpreting has recently extended to SI (Dayter, 2019, 2020). The argument of Henriksen (2007) was the prelude to this development. In the article, Henriksen confirmed the advantage of FS in relieving interpreters’ cognitive loads: having available a large collection of formulas can reduce the effort required for interpreters to produce their output because they can retrieve these formulas as single units from memory. As a result, using formulaic language, which involves combining longer lexical units, frees up cognitive resources that would otherwise be needed for combining shorter lexical items. Henriksen’s argument attributed the advantage of FS in interpreting to the cognitive mechanism of chunking. Although his study was not a databased and large-scale study, it inspired several subsequent studies that aimed to reveal how interpreters efficiently used FS to work (Plevoets and Defrancq, 2018; Li and Halverson, 2020, 2022; Tang and Jiang, 2022). Although not all the studies claimed a cognitive load perspective, they were motivated by the assumption that appropriate use of FS has an alleviating effect. Their investigations of the features of FS in interpreting reveal how interpreters execute the chunking strategy to meet cognitive challenges at work. The contributions and limitations of the studies are discussed in the next section.

### 2.3. Connecting formulaic sequences with cognitive loads in interpreting

Cognitive loads in interpreting have many facets (Seeber, 2011; Köpke and Signorelli, 2012; Injoque-Ricle et al., 2015; Lv and Liang, 2019; Bóna and Bakti, 2020; Chou et al., 2021; Boos et al., 2022; Chang and Chen, 2023; Lin and Liang, 2023). Chen (2017) synthesized potential factors and categorized them into two groups: task and environment characteristics; and interpreter characteristics. An understanding of the construct of cognitive loads in interpreting serves as a useful foundation for exploring the alleviating effects of FS. In Chen’s scheme, task characteristics encompass crucial factors related to the interpreting task, such as interpreting mode (e.g., SI or consecutive interpreting), language pairs (i.e., linguistic differences between the source and target texts), features of speech (e.g., spontaneous speech or interpreted speech), expected response (e.g., a high requirement for accuracy), directionality (L1–L2 or L2–L1 interpreting), etc. Environmental characteristics describe the locations and conditions where interpreters work. For example, interpreters working in noisy environments or with earphones of poor quality may suffer from more cognitive constraints. Interpreters’ characteristics refer to individual differences of interpreters that may affect their performance, including training or working experience, motivation, working memory capacities, etc. More details and examples can be found in Chen’s article.

Corpus-based studies investigating FS in interpreting have focused on factors that broadly fall within the range of those proposed by Chen’s construct (Plevoets and Defrancq, 2018; Li and Halverson, 2020; Wu et al., 2021). Therefore, although not all studies explicitly and directly connect the use of FS to cognitive load management, to varying degrees they attempted to answer this research question.

#### 2.3.1. Connecting FS with task and environment characteristics

A group of three studies have connected FS with interpreting strategies to address language pairs (Li and Halverson, 2020; Wu et al., 2021; Li and Halverson, 2022). These used parallel corpora on the occasions of international political conferences. These events were selected for two main reasons. First, they place high demands on accuracy and timely productions, and thus impose a high cognitive demand on interpreters (Wu et al., 2021). Second, these conferences are often multilingual, an ideal occasion to observe different language pairs. In each of the three studies, the researchers claimed that the linguistic differences between Chinese and English are likely to pose challenges for interpreting. For example, Chinese is monosyllabic, whereas English is multisyllabic; Chinese is a high-context language, while English depends on context much less (Wu et al., 2021). Wu et al. summarized three primary discourse functions of n-grams used by Chinese participants at conferences of the United Nation Security Council (UNSC) and suggested that it would be beneficial to prepare their English counterparts in advance to address cross-linguistic differences. Li and Halverson (2020) analyzed four-word LBs on another political occasion, a Premier Press Conference, through the lens of explicitation. FS help reduce interpreting stress by fulfilling three explicitation functions: simple addition, repetitive addition and quasi-repetitive addition. Based on the same corpus and using the same procedures to retrieve LBs, Li and Halverson (2022) mapped the discourse functions of lexical bundles in interpreted texts with three relationships between source text (ST) and target text (TT), namely, equivalence, shifts, and additions, further exploring how FS can facilitate coordination between ST and TT.

Notably, Li and Halverson (2022, p. 3) realized that “the issue of directionality is of relevance,” and they therefore used data from retour interpreting, i.e., L1–L2 interpreting. Interpreters were supposed to use as many “pat phrases” as possible in retour interpreting (Jones, 1998, p. 122). It is important to consider interpreting directionality because retour interpreting is believed to impose more constraints on interpreters (Liu et al., 2023). Directionality was proposed as a significant factor related to task characteristics that may cause the cognitive loads outlined in Chen’s construct (Chen, 2017, p. 644). However, Li and Halverson did not, in fact, identify differences between the use of FS in L1–L2 interpreting and its use in L2–L1 interpreting. To the best of the author’s knowledge, ever since Jones called for attention to be given to FS in retour interpreting, no empirical evidence has been provided to support the hypothesis that retour interpreting relies more on FS.

An additional relevant factor is speech features. One perspective on this is gained by comparing interpreted and non-interpreted texts. The study of Plevoets and Defrancq (2018) is notable for establishing a directly negative relationship between cognitive loads indicated by the disfluency marker “um(m)” and FS identified in ST and TT. More FS in ST promote interpreters’ anticipation, while those in TT facilitate interpreters’ automatization. In the same study, the alleviating effects of FS were compared in interpreted and non-interpreted texts. Results indicated both texts relied on FS, but non-interpreted texts relied to “a markedly lesser extent” (Plevoets and Defrancq, 2018, p. 22). An additional perspective on this is achieved by incorporating genre or register variations when comparing interpreted and non-interpreted texts. For example, Wu (2021) investigated variations of LBs in texts from interpreters, translators and members of the European Parliament. Texts in the former two categories were translated^2^ texts, but differing in register, while those in the latter category were non-translated texts. Unlike the non-interpreted speeches, the interpreted texts did not show a preference in relation to specific functions of LBs. Stance bundles, referential bundles, and subject-specific bundles were evenly distributed in these kinds of texts. Whereas Wu used a comparable corpus, Biel et al. (2019) used parallel corpora for four genres, namely, legislation, judgments, reports and websites. They found that the length of n-grams, threshold to retrieve them, and most importantly, the genres to which they belonged, correlated to the quantity of LBs in terms of type and token. Therefore, it could not be conclusively determined that either interpreted or non-interpreted texts have a higher level of formulaicity, as this varied depending on multiple factors. Another finding in the study was that there was little overlap in LBs between ST and TT. The authors considered this a “striking finding” because it went against the least effort law proposed by Gile (2009). Without overlap, interpreters had to laboriously trigger FS in the TT rather than working from those present in the ST.

There is a dearth of research on the connection between formulaic language and environmental factors, probably due to ethical problems around exposing interpreters to a detrimental environment, for example, noise or earphones of poor quality. A study of Song (2020) is relevant. In the study, trainee students were divided into controlled and experimental groups. They received ST with an accent to interfere with their comprehension. The experimental group prepared relevant FS in advance, while the other group did not. The experimental group demonstrated more accurate and fluent interpreting performances. A semi-interview with the trainees in the experiment group confirmed the hypothesis that storing FS facilitated interpreters’ anticipation in the listening process, offsetting the accent interference.

#### 2.3.2. Connecting FS with interpreter characteristics

Cognitive loads do not depend solely on the interpreting task or interpreter characteristics: “the amount of workload someone experiences results from the interaction between the situation with its properties and demands and the individual capabilities and resources” (Boos et al., 2022, Introduction). In interpreting studies, interpreter-related factors have attracted researchers’ attention (Young and Stanton, 2001; Macnamara, 2012; Boos et al., 2022; Song and Li, 2023). However, only a few studies have looked in a unified way at interpreter characteristics and their phraseological use (Wang and Huang, 2011, 2013; Tang and Jiang, 2022). In Wang and Huang (2011, 2013), FS were correlated to language proficiency to yield pedagogical implications: students with a higher interpreting proficiency used more FS and used them more appropriately, but interpreting proficiency did not necessarily correlate to erroneous use. As interpreting proficiency improved, the frequency of grammatically incorrect FS decreased while that of incorrect collocations increased. Additionally, a systematic comparison between professional and trainee interpreters’ LBs was made by Tang and Jiang (2022). They described features of LBs more comprehensively in terms of token, type, diversity, structural and interpreting strategy distributions. Results indicated professional interpreters used noun and propositional phrasal frames and the equivalence strategy more frequently than trainees.

Studying FS in the interpreting context is in its early stage. The studies discussed above have considered a variety of factors that may affect the use of FS, including the interpreting strategies adopted to cover the distances in language pairs, the norms of interpreted texts, the directionality, the occasions where interpreting is done, the interpreters’ language proficiency and experiences. They have investigated the quantitative and qualitative features of FS in connection with cognitive loads. These pioneering studies have provided large-scale corpus-based data, supporting the early theoretical proposition of the importance of using FS in interpreting (Jones, 1998; Henriksen, 2007). In addition, they shed light on how interpreters adapt their phraseological use depending on situations. However, they all have one shortcoming in common. They have not considered the interaction of factors. The multitasking nature of interpreting makes it a cognitively complex activity (Stachowiak-Szymczak, 2019, p. 46). Consideration of the interaction of factors would make for a more comprehensive description of cognitive loads in interpreting. It also offers a pathway to deeper understanding of how FS are employed to handle cognitive demands.

#### 2.3.3. A multivariate perspective to study FS in connection with task complexity and interpreting directionality

The current study focuses on SI for three reasons. First, SI is considered “an extreme case of multitasking” (Koshkin et al., 2018, p. 3) in an “extreme condition” (Lv and Liang, 2019, p. 91). Second, scholarly interest in investigating FS in translation has recently extended to SI (Dayter, 2020). Most importantly, SI at UNSC conferences offers a multivariate perspective from which to analyze FS, supplementing the deficiency of previous studies that only considered a single factor.

A comparable corpus of SI in UNSC conferences provides three types of texts and offers an opportunity to interact factors. The first factor is task complexity. Interpreted and non-interpreted texts can be compared. When the delegates use their L1 to make a speech, they produce non-interpreted texts (L1 texts), different to the interpreted speeches from interpreters. The current study adopts a psycholinguistic perspective, borrowing the term task complexity from second language acquisition to distinguish interpreted and non-interpreted texts. Task complexity is a concept used to describe cognitive efforts involved in completing a task. Robinson (2001, p. 29) defined it as a construct, referring to it as “the result of the attentional, memory, reasoning, and other information processing demands imposed by the structure of the task to the language learner.” Skehan (1998) utilized the term “cognitive complexity” to more clearly indicate the relationship between task difficulty and the demand on cognition. The more difficult or complex a task is, the more cognitive resources are required to maintain the speech performance. Non-interpreted texts are a kind of extemporaneous speech, allowing preparation in advance and incorporating cognitive loads primarily from speech production (Bóna and Bakti, 2020). By contrast, the SI interpretation of texts involves the dual tasks of synchronous comprehension and production (Seeber, 2011). Therefore, performing an SI task should be more cognitively demanding than producing speech only. The second factor is interpreting directionality. Interpreters work from their L1 to L2 or vice versa, differing in interpreting directionality. Retour interpreting for L1–L2 texts is considered more cognitively demanding than non-retour interpreting for L2–L1 texts (Chou et al., 2021). However, no empirical evidence has been provided since Jones called for attention to be given to how FS facilitate retour interpreting (Jones, 1998). The current study fills this research gap.

In sum, the interaction of task complexity and directionality increases the cognitive loads from L1 to L2-L1 to L1–L2 texts. These three kinds of text are classified in the current study as texts with low, medium or high cognitive loads. Investigating FS in these texts offers an alternative approach to the univariate perspective of previous studies. Connecting phraseological use with the three degrees of cognitive loads provides hints to “determine whether interpreters deliberately use FS as a compensatory mechanism to offset increasing cognitive loads” (Plevoets and Defrancq, 2018, p. 23).

The current study focuses on p-frames. As mentioned in Section 2.2, p-frames are superior in avoiding overlap in contiguous LBs. Additionally, the slots in p-frames allow for variance, making p-frames more formula of utterances in that a balance between prefabrication and variance is maintained, thus conforming to the idiom and open-choice principles proposed by Sinclair (1991) for FS. P-frames can be of varying lengths. The current study only analyzed four-word p-frames, for two reasons. First, the length of p-frames is not a variable for current consideration. Second, four-word LBs contain structures of three-word ones, and are more frequent than five-word ones (Cortes, 2004; Hyland, 2008).

The effect of cognitive load on the usage of p-frames could be perceived in terms of their quantity and qualitative features. Quantity refers to the number of p-frames and continuous LBs with slots of their corresponding p-frames filled in. Qualitative features include the fixedness and categories of p-frames. Details are presented in the methodological section.

To synthesize the above discussion, the present study proposes the following two research questions:

RQ1: How do various cognitive loads caused by task complexity and directionality affect the quantity of p-frames?
RQ2: How do various cognitive loads caused by task complexity and directionality affect the qualitative features of p-frames?

For the first research question, the hypothesis proposed is that the greater the cognitive loads resulting from a speech, the more p-frames are employed. This hypothesis was made in light of previous studies’ demonstration of the superiority of FS in information processing, as discussed in Section 2.2. Therefore, it is proposed that the non-interpreted texts result in the least cognitive loads and the fewest p-frames. The L1–L2 interpreted texts are expected to result in the greatest cognitive loads and the highest number of p-frames. No hypothesis was made for the second research question since this issue has not been previously explored.

## 3. Research methodology

The current section present information on corpus construction, target p-frames generation with criteria of p-frames selections given in details, measures to ensure inter-reliability of coding and statistics for results and analysis.

### 3.1. Corpus construction

A comparable corpus was constructed for speeches delivered at UNSC conferences, comprising three sub-corpora corresponding to L1, L1–L2, and L2–L1 texts. The L1 corpus consisted of non-interpreted texts in English delivered by L1 speakers (E–E corpus). The L1–L2 corpus consisted of interpreted speeches from Chinese to English by Chinese interpreters (C–E corpus). The L2–L1 corpus consisted of interpreted speeches from Russian to English by English L1 speakers (R–E corpus).

The conference videos were obtained from the official website of the United Nations^3^ using the keyword *Security Council* for searching. One hundred fifty videos were randomly selected and downloaded over the past 3 years (2021–2023). Relevant episodes were extracted. These episodes were transcribed into written form, and the UN documentation website^4^ was used to verify information on proper nouns such as organizations, people, and places. A peer review was conducted to correct spelling mistakes, resulting in a corpus of UNSC texts comprising approximately 41 million words.

The corpus was divided into E–E, R–E, and C–E sub-corpora, with 122,628, 177,835, and 110,638 words, respectively. The C-E corpus was used as a standard, and texts were randomly deleted from the E–E and R–E corpora to match the size of C–E. The three sub-corpora now contained approximately 11 million words each and are comparable in size. Table 1 provides an introduction to the three sub-corpora.

**Table 1:** An introduction to the three corpora.

Corpora	Range of texts	Word count
E–E	142	11, 0679
C-E	160	11,0638
R-E	105	11, 0822

On the one hand, the speeches delivered by delegates at each booth in the UNSC cover similar topics related to global peace and security and have homogenous structures (Wu et al., 2021). Therefore, the range of texts is not expected to impact the results as long as they come from various speakers. Hence, there was no need for the three sub-corpora to have the same range of speeches.

On the other hand, although the three corpora are relatively small, they are specialized and may contain sufficient examples of frequent linguistic features that can “illuminate the comparison between different types of interaction” (Lin, 2013, p. 108). This assumption aligns with Sinclair’s claim that “comparison uncovers differences almost regardless of size” (Sinclair, 2001, p. xii).

### 3.2. P-frame identification

The kfNgram tool (Fletcher, 2011) was used to extract p-frames from the corpus, and criteria were established based on two previous studies (Nekrasova-Beker, 2019; Lu, 2021) to select target p-frames.

Lu (2021) employed automatic generation followed by manual refining to identify target p-frames. KfNgram automatically generated p-frames with two or more variants, consisting of 5–6 words, and used at least three times across three different texts. In the manual refining process, certain groups of p-frames were excluded. For instance, p-frames that were not semantically complete at the clausal boundaries, such as * but we can, were removed. Similarly, p-frames that contained slots that were not semantically consistent, such as a wide range of *, were also excluded. Thirdly, p-frames that were not pedagogically meaningful due to their restricted meaning, such as the United States and the *, were also removed. Finally, p-frames with slots containing too many possibilities of variants and thus revealing no pattern, such as the * of the, were also excluded.

Nekrasova-Beker (2019) employed similar procedures to identify p-frames, but his approach had some key differences compared to Lu′s. Firstly, Beker’s study was based on larger corpora and p-frames that occurred at least 100 times per million words and in at least 75% of texts were used as the threshold for automatic generation. Secondly, only p-frames with one slot and a length of four words were selected as targets. Thirdly, unlike Lu’s study, Nekrasova-Beker included p-frames considered “too broad to reveal no pattern.” Fourthly, p-frames that contained slots that could not be grouped semantically and were excluded by Lu were included in Beker’s study if the filled-in words shared the same part of speech and word class.

The current study adopted criteria for selecting p-frames that combined Lu’s and Beker’s approaches while also fully considering Sinclair’s “open principle” and “idiom principle” (Sinclair, 1991): a target p-frame should strike a balance between fixedness to reflect chunking nature and flexibility to allow for variances in the slots. Ultimately there are five primary criteria:

(1) The number of words in each sub-corpus fell short of the size of the corpus used in the study conducted by Nekrasova-Beker. However, the size of each sub-corpora was double that of Lu’s corpus. As previously mentioned, the range of speeches was not a factor to be considered. Instead, the sole criterion for identifying prominent targets was frequency. A compromise was made to select p-frames that occurred at least ten times. This frequency threshold was deemed sufficient to make the p-frames salient and provide adequate samples for analysis. Moreover, the present study focused solely on 4-word length p-frames.
(2) Too fixed p-frames were excluded. For example, * on the parties for *calls on the parties* and *call on the parties* was not selected because the same lemma *call* is filled into the slot, making the p-frame too restricted and lack of flexibility.
(3) P-frames at the boundary of clauses were excluded. For example, * parties concerned to consists of a main clause of *verb + parties concerned* and *an infinitive clause to + verb*, reflecting two chunking habits, so it should be discarded.
(4) P-frames with morphological, syntactic, or semantic consistency slots were retained for analysis. For example, *nothing to do with* and *having to do with* do not share the same p-frame of *to do with because *nothing* and *having* are not consistent in any of the three aspects. However, the fact that * was included, despite a boundary of clauses in the p-frame, violating the third principle. The variances of its slots belonged to the same syntactic category of a subordinate clause, and the p-frame reflected only one chunking habit of *the fact that + a subordinate clause*.
(5) P-frames with proper nouns, such as the United States *, were excluded as they were deemed pedagogically meaningless. On the other hand, p-frames with domain-specific technical terms, such as on * parties concerned, were included. Such p-frames were considered formulas to facilitate information processing during political occasions.
(6) P-frames that primarily consist of functional words and allow multiple slot possibilities were retained. These p-frames balance idiomaticity and openness, aligning with Sinclair’s principles.

The co-researcher was trained on how to apply the established criteria, and both the author and co-researcher independently selected 100 p-frames at random. The results indicated a high degree of agreement between the two researchers, with a Cohen’s kappa coefficient of 0.80 (*p* < 0.001), indicating substantial agreement. Any discrepancies were discussed and resolved through consensus. Afterward, the researcher proceeded with the remaining p-frames.

The process to ensure inter-rater reliability was repeated in categorizing the selected p-frames grammatically and functionally, resulting in kappa coefficients of 0.85 and 0.80, respectively (*p* < 0.001).

### 3.3. P-frame analysis

The proportions of p-frames and their corresponding LBs from the three sub-corpora were analyzed to measure the quantity of FS. Qualitative features were also considered, including fixedness and grammatical and functional categories of the p-frames. Fixedness was measured using the ratio of type/token (TTR) as an indicator (Nekrasova-Beker, 2019). Token refers to how many times the p-frames occur, while type depends on the number of variations of the slots, with one variation accounting for one type. The smaller the TTR, the more fixed the p-frame is.

For functional categories, the influential scheme proposed by Biber et al. (2004) was used to categorize the p-frames into referential expressions, stance expressions, and discourse organizers. However, the scheme Gray and Biber (2013) proposed was utilized for structural categories. P-frames often have “multiple functions depending on the word that fills the variable slot” (Gray and Biber, 2013, p. 122), which may cause difficulty when categorizing them based on the previous scheme proposed for continuous LBs (Biber et al., 2004). Therefore, Gray and Biber proposed a broader scheme to categorize p-frames structurally. According to their scheme, p-frames can be categorized into verb-based frames, frames with other content words, and function word frames. Table 2 provides a summary of these categories and examples.

**Table 2:** The scheme for categorization (a combination).

Category	Sub-category	Examples
structural category	verb-based frames	I * going to, it is * to, we hope that *, * would like to, will continue to *
	frames with other content words	implementation of the *, the circuit of *, * of the body, settlement of the *, of the * people
	function word frames	on the * hand, on the * of, to the * of, the * in, the of the
functional category	referential expressions	the * of the, the basis of *, an * role in, work of the *, the * way to
	stance expressions	would like to *, it is * to, calls on the *, stands ready to *, we * continue to
	discourse organizers	as shown in *, * shown in figure, in this context *, to * the following, listened * to the

### 3.4. Statistics

Jamovi 2.3.21 (R Core Team, 2021), a computer tool based on R packages, was used for statistical analysis. Two chi-square goodness-of-fit tests were conducted to compare the number of p-frames in the three sub-corpora. An ANOVA test was used to compare the ratio for fixedness. As task complexity and directionality interacted to yield three degrees of cognitive load, categorized as “low, medium, and high,” cognitive load was treated as a categorical variable. Grammatical and functional categories were the other two categorical variables. Two multinomial logistic regression (MLR) tests were employed to determine whether cognitive loads caused grammatical and functional category variations. The MLR is a statistical method used to analyze the relationship between a categorical dependent variable with more than two categories of independent variables. The two MLR tests helped to understand how different cognitive loads contributed to the likelihood of an observation falling into each grammatical or functional category.

## 4. Results and discussion

In this section, comparisons were made of the p-frames across the three sub-corpora to unveil both quantitative (as per RQ1) and qualitative (as per RQ2) variations. The identified variations were subsequently examined in light of the cognitive load induced by task complexity and directionality in SI.

### 4.1. The quantity

Four hundred and five p-frames were identified based on the criteria outlined in the methodology section. Tables 3, 4 display the results of comparisons of frequencies of p-frames and their corresponding continuous LBs, respectively.

**Table 3:** The comparison of the frequency of p-frames.

Proportions-corpora			
Level		Count	Proportion
C-E	Observed	200	0.494
	Expected	135	0.333
E–E	Observed	75	0.185
	Expected	135	0.333
R-E	Observed	130	0.321
	Expected	1,135	0.333

*x*^2^ Goodness of fit		
*x* ^2^	*df*	*p*
58.1	2	< 0.001

**Table 4:** The comparison of the frequency of continuous LBs.

Proportions-corpora			
Level		Count	Proportion
C-E	Observed	4,392	0.478
	Expected	3,064	0.333
E–E	Observed	1,381	0.150
	Expected	3,064	0.333
R-E	Observed	3,420	0.372
	Expected	3,064	0.333

*x*^2^ Goodness of fit		
*x* ^2^	*df*	*p*
1,541	2	<0.001

The test results in Table 3 indicated a significant difference between the observed and expected frequencies of p-frames, with *χ*^2^ (*df* = 2, *N* = 405) = 58.1, *p* < 0.05, indicating a significant difference in the distribution of p-frames across the corpora. Additionally, the test results in Table 4 revealed a significant difference for continuous LBs with slots filled by corresponding p-frames: *χ*^2^(*df* = 2, *N* = 9,192) = 1,541, *p* < 0.05, indicating a significant variation in the distribution of continuous LBs across the corpora. P-frames and their corresponding LBs are linguistic indicators of formulacity, so the following section used the term formulaic sequences (FS) to refer to both. Based on the results in Table 3, 4, it can be concluded that the C-E Corpus employed the most FS, while the E–E Corpus employed the least. The differences were statistically significant.

The finding confirmed the first research question’s first hypothesis: The more cognitive load is incorporated in a task; the more FS are expected to be identified. L1 texts in E–E were supposed to incorporate the least cognitive load and generated the fewest FS (18.5% for p-frames and 15.0% for continuous LBs). Task complexity and directionality interacted to create the highest cognitive load in C-E, which used the most FS (49.4% for p-frames and 47.8% for continuous LBs).

It is noteworthy that task complexity and directionality impacted FS usage to different degrees. When R–E and E–E were compared, there was a sharp difference between their proportions of p-frames (32.1 *vs* 18.5%). However, when R–E and C-E were compared, the difference became smaller (32.1 *vs* 49.4%), indicating that the directionality of interpreting impacted the quantity of FS less than task complexity. Task complexity played a major role in posing cognitive challenges.

The findings reconcile with previous studies. First, they support the assumption that retour interpreting is more cognitively demanding. According to Chmiel (2016), controversial views exist concerning the difficulty of retour interpreting. The first view claims that more effort is required in production, so production in L2 is at a disadvantage. Retour interpreting is more demanding than interpreting into A language. The second one suggests that production and comprehension consume the same effort. The advantage in compression in L1 can offset the disadvantage in production in L2. The current study identified more FS employed in C-E than R-E and provided evidence for the first view. Retour interpreting is more cognitively demanding. Interpreters resorted to the chunking strategy and utilized more FS to maintain acceptable performances.

Second, the findings suggested that task complexity affected language performance. Several empirical studies have examined the relationship between task complexity and language performance regarding lexical and syntactic complexity and fluency(Ong and Zhang, 2010; Johnson, 2017; Abdi Tabari et al., 2023). The present study extended the relationship to the employment of FS: A consensus has been made that increasing task complexity may sacrifice fluency(Skehan, 1998; Robinson, 2001). To maintain fluency in a complex task, interpreters could utilize FS. This finding is consistent with that of Plevoets and Defrancq (2018): the more FS are used, the less disfluent the interpreting speeches are.

### 4.2. The qualitative features

The three sub-corpora were not diverse in fixedness. Significant differences were identified in structural and functional categorization.

#### 4.2.1. Fixedness

**Figure 1:**
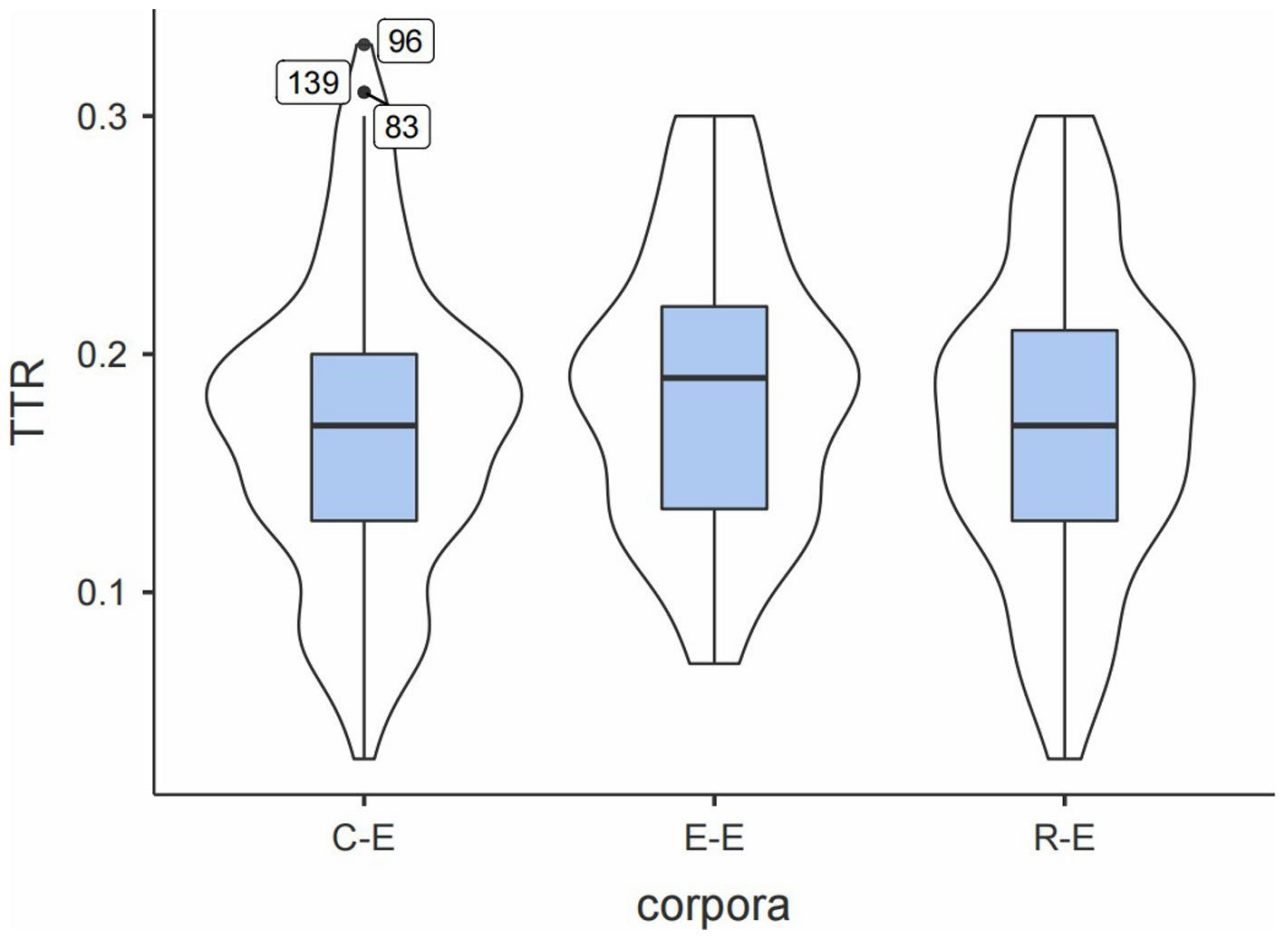
Box plots of ratio of TTRs in the three sub-corpora.

First, the TTRs of the three sub-corpora were visualized through the box plot in Figure 1.

As shown by the three horizontal lines of the box plots, E–E had a slightly higher median value of TTR, indicating more variances. A possible conclusion was that less cognitively demanding tasks permit less fixed p-frames and produce a higher diversity of LBs. Additionally, C-E had the widest interquartile range (IQR), less clustered around the median, indicating a wider range of TTRs. Outliers were highlighted only in C-E. They were * of the security (0.33), solution to the* (0.31), and with the * of the (0.30), all referential expressions. The commonness may be because referential expressions comprised most identified p-frames and were more likely to be encountered. Furthermore, the outliers suggested that the largest TTR was around 0.3, and most p-frames had TTRs less than 0.3. Table 5 displays the top five p-frames with the largest TTRs in each corpus, suggesting that all TTRs indeed had values less than 0.3. Gray and Biber (2013) considered p-frames with TTR less than 0.30 relatively fixed. If this criterion was used, a conclusion was drawn that UNSC speeches tend to employ relatively fixed p-frames.

**Table 5:** Top 5 p-frames ranked by TTR values across the three sub-corpora.

Corpus	P-frames and TTRs
C-E	* of the security (0.33), for the * of (0.29), development in the * (0.29), regional peace and * (0.29), of the * of (0.28).
E–E	this is a * (0.30), the * situation in (0.30), * deeply concerned by (0.30), the * people and (0.30), I’d like to* (0.28).
R-E	* middle east settlement (0.30), that * will be (0.30), under the * of (0.30), of the * we (0.30), * of the situation (0.30), * report on the (0.30).

Examples can be found in Table 5. The ritualized language was indicated by keywords in the p-frames such as *settlement*, *security*, *development*, *peace*, *regional*, *people*, and etc. The keywords revolved around the topic of global security and peace, contributing to the ritualized nature of the FS in UNSC texts.

One-way ANOVA was conducted to determine whether significant differences existed with TTRs in the three sub-corpora. Table 6 displays the results.

**Table 6:** Ratio of type/token (TTR) comparison among the three sub-corpora.

One-Way ANOVA (non-parametric)				
Kruskal–Wallis				
	*x* ^2^	*df*	*p*	*ε* ^2^
TTR	3.78	2	0.151	0.00936

The ANOVA test revealed no significant difference in TTR across the three sun-corpora at a significance level of 0.05 (*χ*^2^ = 3.78, *df* = 2, *p* = 0.151). The effect size (*ε*^2^) was negligible (*ε*^2^ = 0.00936), indicating nearly no difference among the three sub-corpora. That UNSC texts were quite formulaic may be the explanation. Forsyth and Grabowski (2015) believed that relying on more fixed p-frames would indicate more formulaic texts. As mentioned in the results in Table 5, nearly all p-frames in the UNSC corpus were relatively fixed, indicating a heavy reliance on formulaic texts. It seemed that the tendency to use ritualized and formulaic language diminished the influence of cognitive loads. In sum, UNSC texts preferred relatively fixed p-frames regardless of the amount of cognitive load.

#### 4.2.2. Grammatical and functional distribution

As discussed in the literature review section, the cognitive loads increased from L1, L2–L1, to L2–L1 texts. Therefore, E–E had a low cognitive load, R-E had a medium cognitive load, and C-E had a high cognitive load. In this way, the cognitive load became an ordinal variable. Two MLR tests were conducted to determine how various degrees of cognitive loads influenced grammatical and functional choices. They had cognitive load as a predictor. The grammatical category was the dependent variable in the first model. The functional category was the dependent variable in the second model. Tables 7, 8 display the results.

**Table 7:** Multinominal logistic regression of cognitive load and grammatical categories.

Model fit measures			
Model	Deviance	AIC	*R*^2^ McF
1	767	779	0.0212

Model coefficient-grammatical category					
Grammatical category	Predictor	Estimate	SE	*Z*	*p*
	Cognitive load:				
function word frames—frames with other content words	low-medium	−0.634	0.400	−1.583	0.113
	low-high	0.242	0.398	0.608	0.543
	medium-high	0.875	0.290	3.023	0.002※
frames with other content word-verb-based frames	low-medium	−0.478	0.340	−1.405	0.160
	low-high	−0.866	0.314	−2.756	0.006※
	medium-high	−0.388	0.291	−1.332	0.183
function word frames-verb-based frames	low-medium	−1.112	0.439	−2.531	0.011※
	low-high	−0.624	0.435	−1.435	0.151
	medium-high	0.488	0.351	1.390	0.165

**Table 8:** Multinominal logistic regression of cognitive load and functional categories.

Model fit measures			
Model	Deviance	AIC	R^2^McF
2	463	475	0.0391

Model coefficient-functional category					
Functional category	predictor	Estimate	SE	*Z*	*p*
	Cognitive load				
Referential expressions-stance expressions	low-medium	−0.571	0.321	−1.780	0.075
	low-high	−1.221	0.320	−3.818	<0.001※
	medium-high	−0.650	0.294	−2.211	0.027※
Referential expressions-discourse organizers	low-medium	−0.0219	1.237	−0.0177	0.986
	low-high	0.128	1.130	0.113	0.910
	medium-high	0.148	0.875	0.169	0.866
Stance expressions-discourse organizers	low-medium	0.5487	1.253	0.4380	0.661
	low-high	1.349	1.151	1.171	0.241
	medium-high	0.798	0.906	0.881	0.378

Three significant differences existed in the first model. First, the predictor (low to medium cognitive loads) made a difference in the outcome (function-word-based to content-word-based p-frames; *p* < 0.05). The coefficient estimate was 0.875, the standard error (SE) was 0.290, and the *Z*-value was 3.023. The positive coefficient indicated that the transition from medium to high cognitive load (R–E to C–E) was likely to result in the transition from function-word-based bundles to content-word-based bundles. Specifically, 66.0% of p-frames in C-E were p-frames with other content words, and 15% were function-word-based p-frames; 58.3% were p-frames with other content words in R-E and 27.7% were function-word-based p-frames.

Second, the predictor (low to high cognitive loads) made a difference in the outcome (frames with other content words to verb-based frames; *p* < 0.05). The coefficient estimate was − 0.866, the standard error (SE) was 0.314, and the *Z*-value was −2.756. Third, the predictor (low to medium cognitive loads) made a difference in the outcome (function word frames-verb-based frames; *p* < 0.05). The coefficient estimate was −1.112, the standard error (SE) was 0.439, and the *Z*-value was −2.531. The negative coefficients in the second and the third results suggested that increasing cognitive load from low to medium and low to high levels reduced the likelihood of using verb-based frames. In C–E and R–E, verb-based p-frames had 19.0 and 21.5%, respectively, whereas the proportion increased to 34.7% in E–E.

In sum, two findings were yielded in the first model. First, directionality made a difference. To cope with cognitive loads in interpreting, interpreters in L2–L1 interpreting preferred function word bundles, while those in L1–L2 interpreting preferred frames with other content words to offset the disadvantage in retour interpreting. The top five frequent function word bundles in L2–L1 texts were the * of the (419)^5^, in the * of (126), on the * of (103), of the * of (69), the * in the (64). Among them, the * of the had far more occurrences than others. The top five frequent frames with other content words in L1-L2 interpreting were peace and * in (49), implementation of the * (35), * settlement of the (43), * and stability in (43) and of the international *(43). One possible explanation for the tendency is that content words are often more informative than function words. Storing more informative words in advance while producing less informative words on the spot reduces the cognitive loads of online processing. Interpreters can adapt their chunking strategy by prefabricating more frames with content words to manage a denser cognitive load when they have to do interpreting from A to B language.

Second, task complexity influenced the usage of verb-based frames. Texts in a simple task in E–E were more likely to incorporate verb-based bundles. The frequently used verb-based p-frames included *we call on* *, we * continue to, will continue to *, look forward to *, we call * the, we are * to, deeply concerned * the, I’d like to *, call on all * etc. The reason why non-interpreted texts employed more verb-based p-frames was provided in investigating the correlation between cognitive loads and functional categories in the second model.

Two significant differences existed in the second model. First, the transition from low to high cognitive load (E–E to C–E) increased the likelihood of a shift from stance to referential expressions (*p* < 0.01). The coefficient estimate was 1.221, the SE was 0.320, and the *Z*-value was 3.818. Second, the transition from medium to high cognitive load (R–E to C–E) resulted in a shift from stance to referential expressions (*p* = 0.027). The coefficient estimate was 0.650, the SE was 0.294, and the *Z*-value was 2.211. A combination of the two findings indicated that increasing cognitive loads increases the chance of using referential expressions, i.e., decreasing the chance of using stance expressions. Specifically, E–E had 34.7% of stance expressions, R–E had 23.0%, and C–E with the highest cognitive loads had 13.5%. Stance expressions are commonly used to comment or show attitudes (Biber et al., 2004). They were primarily used to show attitudes in E–E corpus with non-interpreted texts. The following are examples of two p-frames we*continue to and look forward to *. They expressed the speakers’ attitudes toward their plans.

*We will continue to be a champion for Afghanistan’s women and girls*. (9118^th^ meeting).

*We must continue to demand and defend the right of Afghan women…*(8886^th^ meeting).

*We look forward to working closely with our fellow council members…* (8918^th^ meeting).

*The United States look forward to those conversations*. (8873^rd^ meeting).

Other examples include will continue to *, we are * to, * concerned about the, I’d like to *, call on all *, urge * parties to, it is * that, also * to thank, etc. When confronted with cognitively demanding tasks, the interpreters seemed to neglect using stance expressions to make comments or show attitudes.

An issue to be resolved in the first model was why interpreters in retour interpreting (C–E corpus) tended to use frames with other content words rather than verbs. The correlation between grammatical and functional categories explained for it. As presented in Table 9, a Chi-square test of Independence was performed to assess the relationship between grammatical and functional categories. The two variables had a significant relationship: *χ*^2^ (4, 405) = 300; *p* < 0.001. Cramer’s V indicated an effect size larger than 0.5 (*V* = 0.608). For the function of stance expressions, most of them centered on verb-based p-frames. For example, all the examples raised for stance expressions above (we call on*, will continue to *, look forward to *, we are * to, * concerned about the, I’d like to *, call on all *, urge * parties to, it is * that, also * to thank…) are verb-based p-frames. Interpreters confronted with medium or high cognitive load were less likely to use stance expressions, and that may be why they did not have to rely on verb-based p-frames, which was the explanation for the second unsettled issue in the first model.

**Table 9:** The correlation between grammatical and functional categories.

Contingency tables				
Grammatical category	Functional category			
	Discourse organizers	Referential expressions	Stance expressions	Total
Frames with other content words	2	232	2	236
Function word frames	4	69	4	77
Verb-based frames	1	14	77	92
Total	7	315	83	405

*x*^2^ Tests			
Value		*df*	*p*
xN	300	4	<0.001
	405		

Nominal	
	Value
Phi-coefficient Cramer’s *V*	NaN 0.608

## 5. Conclusion

This study argued that multiple factors contribute to cognitive loads in simultaneous interpreting, particularly in conference interpreting (CI). Task complexity and directionality are crucial factors influencing cognitive loads in CI. This study investigated the empirical evidence on how interpreters employed the chunking strategy to cope with cognitive loads in CI caused by these two factors.

The study examined the variations in p-frames among L1, L1–L2, and L2–L1 texts in a comparable corpus of UNSC conferences. The results showed that task complexity and directionality worked together to cause various degrees of cognitive loads, which is positively correlated with the number of identified p-frames. Retour interpreting (L1–L2 texts) used the most LBs, while L1 texts used the least. Therefore, the study concludes that the more cognitive load involved in a text for CI, the more formulaic sequences (FS) are utilized.

The study also examined the relationship between cognitive loads and structures and functions of the p-frames. First, cognitive loads made no difference in p-frames’ fixedness across the three texts. All three texts tended to employ relatively fixed p-frames to produce formulaic texts. Second, directionality affected the structural choices of interpreters. Retour interpreting preferred p-frames with other content words (rather than verbs), while L2–L1 interpreting tended to use function word p-frames. Interpreters in more cognitively demanding retour interpreting employed the chunking strategy through prefabricating p-frames with other content words to prepare the information-loaded items in advance. The preference over content-word-based p-frames may help the interpreters “render all of the information with precision” (Wu et al., 2021, p. 508) and save efforts to meet “challenges of interpreting from Chinese to English due to the differences between the two languages” (Wu et al., 2021, p. 503). Finally, task complexity affected the functional categories. Texts in simple tasks with the least cognitive load preferred stance expressions. The delegates could comment and express their attitudes more when they did not have to be busily engaged with the cognitive loads. There was a high correlation between the structure and function of p-frames. Most stance expressions were in the structure of verb-based p-frames. In cognitive-loaded L1–L2 interpreting tasks, declaring stances was not a top priority, so there is less need for verb-based p-frames.

Several limitations should be acknowledged. First, two multinominal regression models yielded small values of *R*^2^ (0.0212 and 0.0391), indicating that the models explained only a small proportion of the observed variances. Other factors may have resulted in variations among the three speeches, such as the different distances between source and target texts in C–E and R–E sub-corpora, informativeness of the source texts, and most importantly, the interpreters’ language and cultural backgrounds. For example, L2 speakers may depend on explicit grammatical rules rather than functional-based bundles to facilitate grammar accuracy, so they do not prefer the function word frames. On the contrary, native speakers have a more intuitive understanding of grammatical structures. The implicit grammatical knowledge of the native speakers takes the form of prefabricated chunks. Second, the current study hesitated in deciding on p-frames consisting of proper nouns and excluded all of them for adherence to the fifth criterion of p-frame selection. Although the United States * is meaningless, a p-frame like China hopes that * makes sense. Future studies should aim to identify additional criteria for manual selection and provide more detailed guidance on applying these criteria effectively.

## Data availability statement

The original contributions presented in the study are included in the article/Supplementary material, further inquiries can be directed to the corresponding author.

## Author contributions

DH was responsible for conceptualization, wrote the manuscript, and undertook the statistical analyses. FL was responsible for corpus construction and coded as a co-researcher, HG was responsible for conceptualization, organized the research team and revised the manuscript. All authors contributed to the article and approved the submitted version.

## Funding

This work was supported by 2021 Guangdong Education Science Planning Project “The Design and Application of Shared Intelligent Corpus of Interpreting Teaching in the Era of Technology Empowerment” (grant no.: 2021GXJK198).

## Conflict of interest

The authors declare that the research was conducted in the absence of any commercial or financial relationships that could be construed as a potential conflict of interest.

## Publisher’s note

All claims expressed in this article are solely those of the authors and do not necessarily represent those of their affiliated organizations, or those of the publisher, the editors and the reviewers. Any product that may be evaluated in this article, or claim that may be made by its manufacturer, is not guaranteed or endorsed by the publisher.

## Footnotes

^1^Speech and texts both refer to discourses for interpreting. The term text is used by the current study to be consistent with traditional terminology of source and target texts.

^2^The “translated” is used in a broad sense to describe translated and interpreted texts.

^3^https://media.un.org/en/webtv/

^4^https://research.un.org/en/docs/sc/quick/meetings/2023

^5^The numbers in brackets refer to tokens.

## References

[ref1] Abdi Tabari M. Lu X. Wang Y. (2023). The effects of task complexity on lexical complexity in L2 writing: an exploratory study. System (Linköping) 114:103021. doi: 10.1016/j.system.2023.103021

[ref2] Aston G. (2018). “Acquiring the language of interpreters: a corpus-based approach,” in Making Way in Corpus-based Interpreting Studies, New Frontiers in Translation Studies, eds. M. Russo et al. (Springer Nature Singapore Pte Ltd), 83–96.

[ref3] Biber D. Barbieri F. (2007). Lexical bundles in university spoken and written registers. Anatomie et physiologie à l'usage des infirmières 26, 263–286. doi: 10.1016/j.esp.2006.08.003

[ref4] Biber D. Conrad S. Cortes V. (2004). If you look at … lexical bundles in university teaching and textbooks. Appl. Linguis. 25, 371–405. doi: 10.1093/applin/25.3.371

[ref5] Biel L. Kozbial D. Wasilewska K. (2019). The formulaicity of translations across EU institutional genres a corpus-driven analysis of lexical bundles in translated and non-translated language. Translation Spaces 8, 67–92. doi: 10.1075/ts.00013.bie

[ref6] Bóna J. Bakti M. (2020). The effect of cognitive load on temporal and disfluency patterns of speech: evidence from consecutive interpreting and sight translation. Target 32, 482–506. doi: 10.1075/target.19041.bon

[ref7] Bonhage C. E. Fiebach C. J. Bahlmann J. Mueller J. L. (2014). Brain signature of working memory for sentence structure: enriched encoding and facilitated maintenance. J. Cogn. Neurosci. 26, 1654–1671. doi: 10.1162/jocn_a_00566, PMID: 24405186

[ref8] Boos M. Kobi M. Elmer S. Jäncke L. (2022). The influence of experience on cognitive load during simultaneous interpretation. Brain Lang. 234:105185. doi: 10.1016/j.bandl.2022.105185, PMID: 36130466

[ref9] Chang V. C.-Y. Chen I. F. (2023). Translation directionality and the inhibitory control model: a machine learning approach to an eye-tracking study. Front. Psychol. 14:1196910. doi: 10.3389/fpsyg.2023.1196910, PMID: 37205087 PMC10187886

[ref10] Chase W. G. Simon H. A. (1973). Perception in chess. Cogn. Psychol. 4, 55–81. doi: 10.1016/0010-0285(73)90004-2

[ref11] Chen S. (2017). The construct of cognitive load in interpreting and its measurement. Perspect. Stud. Transl. 25, 640–657. doi: 10.1080/0907676x.2016.1278026

[ref12] Chmiel A. (2016). Directionality and context effects in word translation tasks performed by conference interpreters. Poznan Stud. Contemp. Linguist. 52, 269–295. doi: 10.1515/psicl-2016-0010

[ref13] Chou I. Liu K. Zhao N. (2021). Effects of directionality on interpreting performance: Evidence from interpreting between Chinese and English by trainee interpreters. Front. Psychol. 12. doi: 10.3389/fpsyg.2021.781610 PMC8661131 34899532

[ref14] Cortes V. (2004). Lexical bundles in published and student disciplinary writing: examples from history and biology. English Specif. Purp. (New York, N.Y.) 23, 397–423. doi: 10.1016/j.esp.2003.12.001

[ref15] Cowan N. (2001). The magical number 4 in short-term memory: a reconsideration of mental storage capacity. Behav. Brain Sci. 24, 87–114. doi: 10.1017/s0140525x01003922, PMID: 11515286

[ref16] Dayter D. (2019). “Collocations in non-interpreted and simultaneously interpreted English: a corpus study,” in New empirical perspectives on translation and interpreting. ed. L. Vandevoorde (London: Routledge), 67–91.

[ref17] Dayter D. (2020). Strategies in a corpus of simultaneous interpreting. Effects of directionality, phraseological richness, and position in speech event. Meta (Montréal) 65, 594–617. doi: 10.7202/1077405ar

[ref18] Diedrichsen J. Kornysheva K. (2015). Motor skill learning between selection and execution. Trends Cogn. Sci. 19, 227–233. doi: 10.1016/j.tics.2015.02.003, PMID: 25746123 PMC5617110

[ref19] Ellis N. C. (2003). Constructions, chunking, and connectionism: The emergence of second language structure. Oxford: Blackwell Publishing Ltd, 63–103.

[ref20] Fletcher W. H. (2011). KfNgram. Annapolis, MD: USNA.

[ref21] Forsyth R. S. Grabowski Ł. (2015). Is there a formula for formulaic language? Poznan Stud. Contemp. Linguist. 51, 511–549. doi: 10.1515/psicl-2015-0019

[ref23] Gile D. (2008). “Conference interpreting, historical and cognitive perspectives,” in Routledge Encyclopedia of translation studies second edition. eds. B. Mona and G. Saldanha (London, New York: Routledge), 51–56.

[ref22] Gile D. (2009). Basic Concepts and Models for Interpreter and Translator Training. Amsterdam: John Benjamins Publishing Company.

[ref24] Gobet F. Lane P. C. R. Croker S. Cheng P. C-H. Jones G. Oliver L.. (2001). Chunking mechanisms in human learning. Trends Cogn. Sci. 5, 236–243. doi: 10.1016/s1364-6613(00)01662-4 11390294

[ref25] Gray B. Biber D. (2013). Lexical frames in academic prose and conversation. Int. J. Corpus Linguist. 18, 109–136. doi: 10.1075/ijcl.18.1.08gra

[ref26] Henriksen L. (2007). The song in the booth: formulaic interpreting and oral textualisation. Interpreting 9, 1–20. doi: 10.1075/intp.9.1.02hen

[ref27] Hyland K. (2008). As can be seen: lexical bundles and disciplinary variation. English Specific Purposes (New York, N.Y.) 27, 4–21. doi: 10.1016/j.esp.2007.06.001

[ref28] Ilg G. (1985). Expressions. Meta 30, 65–67. doi: 10.7202/003165ar

[ref29] Injoque-Ricle I. Barreyro J. P. Formoso J. Jaichenco V. I. (2015). Expertise, working memory and articulatory suppression effect: their relation with simultaneous interpreting performance. Adv. Cogn. Psychol. 11, 56–63. doi: 10.5709/acp-0171-1, PMID: 26207153 PMC4511188

[ref30] Johnson M. D. (2017). Cognitive task complexity and L2 written syntactic complexity, accuracy, lexical complexity, and fluency: a research synthesis and meta-analysis. J. Second. Lang. Writ. 37, 13–38. doi: 10.1016/j.jslw.2017.06.001

[ref31] Jones R. (1998). Conference Interpreting Explained. Manchester: St. Jerome Pub.

[ref32] Köpke B. Signorelli T. M. (2012). Methodological aspects of working memory assessment in simultaneous interpreters. Int. J. Bilingual. 16, 183–197. doi: 10.1177/1367006911402981

[ref33] Koshkin R. Shtyrov Y. Myachykov A. Ossadtchi A. (2018). Testing the efforts model of simultaneous interpreting: an ERP study. PLoS One 13:e0206129. doi: 10.1371/journal.pone.0206129, PMID: 30356337 PMC6200263

[ref34] Kuiper K. Haggo D. (1984). Livestock auctions, oral poetry, and ordinary language. Lang. Soc. 13, 205–234. doi: 10.1017/s0047404500010381

[ref35] Li L. Franken M. Wu S. (2020). Bundle-driven move analysis: sentence initial lexical bundles in PhD abstracts. English Specific Purposes (New York, N.Y.) 60, 85–97. doi: 10.1016/j.esp.2020.04.006

[ref36] Li Y. Halverson S. L. (2020). A corpus-based exploration into lexical bundles in interpreting. Across Lang. Cult. 21, 1–22. doi: 10.1556/084.2020.00001

[ref37] Li Y. Halverson S. L. (2022). Lexical bundles in formulaic interpreting a corpus-based descriptive exploration. Transl. Interpreting Stud. doi: 10.1075/tis.19037.li

[ref38] Liang J. Lv Q. Liu Y. (2019). Quantifying interpreting types: language sequence mirrors cognitive load minimization in interpreting tasks. Front. Psychol. 10:285. doi: 10.3389/fpsyg.2019.00285, PMID: 30833918 PMC6387939

[ref39] Lin Y.-L. (2013). “Discourse functions of recurrent multi-word sequences in online and spoken intercultural communication,” in Yearbook of Corpus Linguistics and Pragmatics 2013. ed. J. Romero-Trillo (Dordrecht: Springer), 105–129.

[ref40] Lin P. (2022). Developing an intelligent tool for computer-assisted formulaic language learning from you tube videos. ReCALL (Cambridge, England) 34, 185–200. doi: 10.1017/s0958344021000252

[ref41] Lin Y. Liang J. (2023). Informativeness across interpreting types: implications for language shifts under cognitive load. Entropy (Basel, Switzerland) 25:243. doi: 10.3390/e25020243, PMID: 36832610 PMC9955845

[ref42] Liu Y. Cheung A. K. F. Liu K. (2023). Syntactic complexity of interpreted, L2 and L1 speech: a constrained language perspective. Lingua 286:103509. doi: 10.1016/j.lingua.2023.103509

[ref43] Lu X. (2021). Rhetorical and phraseological features of research article introductions: variation among five social science disciplines. System (Linköping) 100:102543. doi: 10.1016/j.system.2021.102543

[ref44] Lv Q. Liang J. (2019). Is consecutive interpreting easier than simultaneous interpreting?—a corpus-based study of lexical simplification in interpretation. Perspect. Stud. Translatol. 27, 91–106. doi: 10.1080/0907676x.2018.1498531

[ref45] Ma X. Li D. (2021). A cognitive investigation of ‘chunking’ and ‘reordering’ for coping with word-order asymmetry in English-to-Chinese sight translation: Evidence from an eye-tracking study. Interpreting 23, 192–221. doi: 10.1075/intp.00057.ma

[ref46] Macnamara B. (2012). Interpreter cognitive aptitudes. J. Interpret. 19:1.

[ref47] Miller G. A. (1994). The magical number seven, plus or minus two: some limits on our capacity for processing information. Psychol. Rev. 101, 343–352. doi: 10.1037//0033-295x.101.2.343, PMID: 8022966

[ref48] Moon R. (1997). “Vocabulary connections: multi-word items in English,” in Vocabulary: Description, Acquisition and Pedagogy. eds. N. Schmitt and M. McCarthy (Cambridge: Cambridge University Press), 105–129.

[ref49] Nattinger J. R. DeCarrico J. S. (1992). Lexical Phrases and Language Teaching. Oxford: Oxford University Press.

[ref50] Nekrasova-Beker T. M. (2019). Discipline-specific use of language patterns in engineering: a comparison of published pedagogical materials. J. Engl. Acad. Purp. 41:100774. doi: 10.1016/j.jeap.2019.100774

[ref51] O'Keeffe A. McCarthy M. Carter R. (2007). From Corpus to Classroom: Language Use and Language Teaching. Cambridge: Cambridge University Press.

[ref52] Ong J. Zhang L. J. (2010). Effects of task complexity on the fluency and lexical complexity in EFL students’ argumentative writing. J. Second. Lang. Writ. 19, 218–233. doi: 10.1016/j.jslw.2010.10.003

[ref53] Pawley A. Syder F. H. (1983). “Two puzzles for linguistic theory: nativelike selection and nativelike fluency,” in Language and Communication. eds. J. C. Richards and R. V. Schmidt (London: Longman), 203–239.

[ref54] Plevoets K. Defrancq B. (2018). The cognitive load of interpreters in the European Parliament a corpus-based study of predictors for the disfluency uh(m). Interpreting 20, 1–32. doi: 10.1075/intp.00001.ple

[ref55] R Core Team (2021). R: A language and environment for statistical computing. (Version 4.1) [Computer software]. Available at: https://cran.r-project.org. (R packages retrieved from MRAN snapshot 2022-01-01).

[ref56] Robinson P. (2001). Task complexity, task difficulty, and task production: exploring interactions in a componential framework. Appl. Linguis. 22, 27–57. doi: 10.1093/applin/22.1.27

[ref57] Schmitt N. Carter R. (2004). “Formulaic sequences in action: an introduction” in Formulaic Sequences. ed. Schmitt N. (Amsterdam: John)

[ref58] Seeber K. G. (2011). Cognitive load in simultaneous interpreting: existing theories—new models. Interpreting 13, 176–204. doi: 10.1075/intp.13.2.02see

[ref59] Seeber K. G. (2013). Cognitive load in simultaneous interpreting measures and methods. Target 25, 18–32. doi: 10.1075/target.25.1.03see

[ref60] Segawa J. Masapollo M. Tong M. Smith D. J. Guenther F. H. (2019). Chunking of phonological units in speech sequencing. Brain Lang. 195:104636. doi: 10.1016/j.bandl.2019.05.001, PMID: 31202179 PMC6686190

[ref61] Sinclair J. (1991). Corpus, Concordance, Collocation. Oxford: Oxford University Press.

[ref62] Sinclair J. (2001). “Preface to small corpus studies and ELT,” in Small Corpus Studies and ELT: Theory and Practice. eds. M. Ghadessy, et al. (Amsterdam: John Benjamins), 7–15.

[ref63] Skehan P. (1998). A Cognitive Approach to Language Learning. Oxford: Oxford University Press.

[ref64] Song J. (2020). Relieving effects of prefabricated chunks in conference interpreting from English to Chinese in an ELF context. Asia Pacific Trans. Intercultural Studies 7, 214–229. doi: 10.1080/23306343.2020.1756174

[ref65] Song S. Li D. (2023). Aptitude for interpreting: the predictive value of cognitive fluency. Interpret. Transl. Train. 17, 155–172. doi: 10.1080/1750399x.2023.2170045

[ref66] Stachowiak-Szymczak K. (2019). Eye Movements and Gestures in Simultaneous and Consecutive Interpreting. Cham: Springer.

[ref67] Tang F. Jiang S. (2022). Four-word lexical bundles in Chinese-English consecutive interpreting—a comparative study between professionals and trainees. Front. Psychol. 13:1005532. doi: 10.3389/fpsyg.2022.1005532, PMID: 36312165 PMC9611200

[ref68] Thompson J. J. McColeman C. M. Stepanova E. R. Blair M. R. (2017). Using video game telemetry data to research motor chunking, action latencies, and complex cognitive-motor skill learning. Top. Cogn. Sci. 9, 467–484. doi: 10.1111/tops.12254, PMID: 28176483

[ref69] Thornton M. A. Conway A. R. A. (2013). Working memory for social information: chunking or domain-specific buffer? NeuroImage (Orlando, Fla.) 70, 233–239. doi: 10.1016/j.neuroimage.2012.12.063, PMID: 23298748

[ref70] Wang W. Y. Huang Y. (2011). The use of chunks in Chinese-English consecutive interpreting of senior students majoring in English. Foreign Lang. Teach. 5, 73–77.

[ref71] Wang W. Y. Huang Y. (2013). The use of chunksand the quality of oral interpretation: an empirical study. Technol. Enhanced Foreign Lang. 4, 28–35. doi: 10.3969/j.issn.1001-5795.2013.04.005

[ref72] Wood D. (2010). Lexical Clusters in an EAP Textbook Corpus. London/New York: Continuum.

[ref73] Wray A. (2000). Formulaic sequences in second language teaching: principle and practice. Appl. Linguis. 21, 463–489. doi: 10.1093/applin/21.4.463

[ref74] Wray A. (2002). Formulaic Language and the Lexicon. Cambridge [England]: Cambridge University Press.

[ref75] Wu Y. (2021). Lexical bundles in English EU parliamentary discourse-variation across interpreted, translated, and spoken registers. Compil. Transl. Rev. 14. doi: 10.29912/CTR.202109_14(2).000237-86

[ref76] Wu B. Cheung A. K. F. Xing J. (2021). Learning Chinese political formulaic phraseology from a self-built bilingual United Nations security council corpus: a pilot study. Babel 67, 500–521. doi: 10.1075/babel.00233.wu

[ref77] Wu Y. Liao P. (2018). Re-conceptualising interpreting strategies for teaching interpretation into a B language. Interpret. Transl. Train. 12, 188–206. doi: 10.1080/1750399x.2018.1451952

[ref78] Yang L. (2018). Effects of three tasks on interpreting fluency. Interpret. Transl. Train. 12, 423–443. doi: 10.1080/1750399x.2018.1540211

[ref79] Young M. S. Stanton N. A. (2001). Mental workload: theory, measurement, and application. Int. Encyclopedia Ergonom. Human Factors 1, 507–509.

