# Corrigendum: Chunking in simultaneous interpreting: the impact of task complexity and translation directionality on lexical bundles

**DOI:** 10.3389/fpsyg.2023.1330110

**Published:** 2023-11-08

**Authors:** Dan Feng Huang, Fang Li, Hang Guo

**Affiliations:** ^1^School of Foreign Languages, Guangzhou College of Commerce, Guangzhou, Guangdong, China; ^2^School of Foreign Languages, Sun Yat-Sen University, Guangzhou, Guangdong, China; ^3^International College, Guangzhou College of Commerce, Guangzhou, Guangdong, China

**Keywords:** simultaneous interpreting, lexical bundles, cognitive load, task complexity, directionality

In the published article, there was an error in affiliation(s) 1 and 3. For affiliation 1, instead of “Faculty of Foreign Language Study, Guangzhou Commerce College, Guangzhou, Guangdong, China”, it should be “School of Foreign Languages, Guangzhou College of Commerce, Guangzhou, Guangdong, China”. For affiliation 3, instead of “International College, Guangzhou Commerce College, Guangzhou, Guangdong, China” it should be “International College, Guangzhou College of Commerce, Guangzhou, Guangdong, China”.

The authors apologize for these errors and state that this does not change the scientific conclusions of the article in any way. The original article has been updated.

